# Wine Fining with Plant Proteins

**DOI:** 10.3390/molecules24112186

**Published:** 2019-06-11

**Authors:** Matteo Marangon, Simone Vincenzi, Andrea Curioni

**Affiliations:** 1 Department of Agronomy, Food, Natural Resources, Animals and Environment (DAFNAE), University of Padova, Viale dell’Università 16, 35020 Padova, Italy; matteo.marangon@unipd.it (M.M.); simone.vincenzi@unipd.it (S.V.); 2 Centre for Research in Viticulture and Enology (CIRVE), Viale XXVIII Aprile 14, 31015 Conegliano, Italy

**Keywords:** wine, plant proteins, allergens, phenolics, fining

## Abstract

Fining treatments involve the addition of a substance or a mixture to wine, and are generally carried out in order to clarify, stabilize or modify the wine’s organoleptic characteristics. Usually these fining agents will bind the target compound(s) to form insoluble aggregates that are subsequently removed from the wine. The main reasons to perform wine fining treatments are to carry out wine clarification, stabilization and to remove phenolic compounds imparting unwanted sensory characteristics on the wine, which is an operation that often relies on the use of animal proteins, such as casein, gelatin, egg and fish proteins. However, due to the allergenic potential of these animal proteins, there is an increasing interest in developing alternative solutions including the use of fining proteins extracted from plants (e.g., proteins from cereals, grape seeds, potatoes, legumes, etc.), and non-proteinaceous plant-based substances (e.g., cell wall polysaccharides and pomace materials). In this article, the state of the art alternative fining agents of plant origins are reviewed for the first time, including considerations of their organoleptic and technological effects on wine, and of the allergenic risks that they can pose for consumers.

## 1. Introduction

Fining is a widely used oenological practice that involves a substance or a mixture being added in order to clarify, stabilize or modify the organoleptic characteristics of the wine. Usually the fining agents bind the target compound(s) to form insoluble aggregates that are subsequently removed from the wine [1]. Given that a common reason to carry out wine fining treatments is to remove phenolic compounds, and that tannins have a high tendency to bind proteins [1], many of the fining agents used are of a proteinaceous origin. A wide range of proteins have been proposed and are currently used as fining agents in winemaking. These can be broadly classified as: i) animal proteins, generally obtained from collagen (e.g., bovine and porcine gelatin) [2,3], egg (e.g., ovalbumin), milk (e.g., caseinates), fish (e.g., isinglass) [4], and, more recently, ii) plant proteins, obtained from cereals (e.g., wheat gluten, corn zeins) [5,6,7,8,9], legumes (e.g., pea, soy and lentil proteins) [10], potatoes (e.g., patatin) [11,12], seaweeds (e.g., spirulina) [13], and grape seeds [14,15]. These proteins have been extensively studied to assess their fining efficacy and their impact on wine sensory characteristics, clarity, stability, and composition. Moreover, since many of these proteins can have an allergenic potential, some studies have evaluated the possibility that residual fining agents can remain in treated wines, thus posing risks for allergic consumers [16]. 

**Figure 1:**
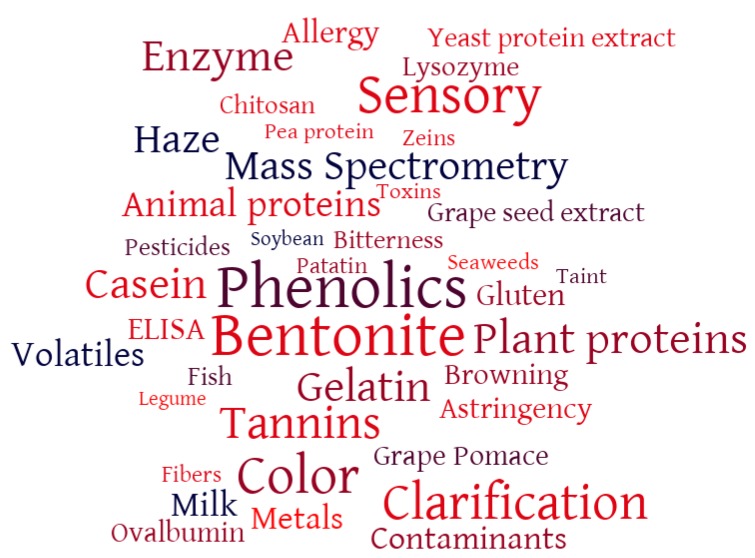
Wordcloud representation of the main key words occurrences in publications found during a Scopus search executed on the 26th of February 2019 (n = 302, publication range 1905–2019). Created with worditout.com. The total number of mentions was 2273, with each word mentioned as follows (number of occurrences enclosed in parentheses): Phenolics (169), Bentonite (161), Sensory (140), Color (135), Clarification (122), Tannins (110), Enzyme (104), Plant proteins (102), Gelatin (97), Casein (95), Mass Spectrometry (89), Haze (86), Animal proteins (70), Volatiles (66), Milk (61), Contaminants (55), Metals (52), Gluten (50), ELISA (48), Allergy (47), Astringency (43), Browning (39), Ovalbumin (36), Grape Pomace (35), Lysozyme (31), Yeast protein extract (29), Grape seed extract (28), Chitosan (25), Fish (24), Bitterness (23), Pesticides (17), Fibers (16), Patatin (15), Zeins (12), Pea protein (9), Toxins (9), Taint (9), Seaweeds (7), Legume (4), Soybean (3).

Interestingly, from an analysis of the publication outputs of researchers operating in this field, it was found that 302 articles on wine fining, published in the period 1905–2019, are currently listed on the Scopus database. From the 302 articles, key words were searched in order to highlight the main areas of interest of research when investigating wine fining. A total of 40 key words were selected to create a wordcloud (Figure 1).

The most repeated keyword is “phenolics”, suggesting that most of the investigations in this field are connected with the phenolic content and composition of wines after fining treatments. Indeed, 6 of the other keywords considered (sensory, color, tannins, astringency, browning, and bitterness) can be related to wine phenolics, accounting for 27.3% of the total mentions (620 out of 2273). The second most represented category of keywords relates to wine clarification (bentonite, clarification, haze, enzymes, 20.8% of mentions), followed by words indicating animal proteins (gelatin, casein, animal proteins, milk, ovalbumin, lysozyme, fish, 18.2% of mentions), plant materials and plant proteins (plant proteins, gluten, grape pomace, grape seed extract, patatin, zeins, pea protein, zeins, legume, soybean, 9.5% of mentions), wine sensory characteristics (sensory and volatiles, 9.1% of mentions), non-protein fining agents (bentonite, chitosan, fibers, 8.9% of mentions), methods of detection or allergy (mass spectrometry, ELISA, allergy, 8.1% of mentions), and residues in wine (contaminants, metals, pesticides, toxins, taints, 6.2% of mentions). This analysis indicates that attention is being paid towards the role played by wine fining on the modifications of phenolic content and profile of the wine, but also on its sensorial properties. Moreover, investigations have been carried out on proteinaceous fining agents, mainly from animal and plant sources, while there is also a certain interest in developing methods to detect protein residues with allergenic potential.

This article is the first that is specifically focused on plant proteins as wine fining agents, an area of wine production that has seen constant development in the past couple of decades and as such is in need of being reviewed with the aim of bringing together past work and proposing future directions of development. In particular, the state of the art of the protein fining agents of non-animal origin is reviewed, including considerations of their effects on the organoleptic and technological properties of treated wines, as well as of the allergenic risks that they could pose for consumers. Moreover, the general steps needed to develop a novel, effective and safe plant-based processing aid will be discussed.

## 2. Wine Fining with Plant Proteins 

At the end of the 21st century, the spread of Bovine Spongiform Encephalopathy led to a review of food production practices. Winemaking was also affected by this review, as several of the fining agents used at the time were of animal origin. These agents included bovine proteins, which were then abandoned as a precaution. As a consequence nowadays porcine gelatin is probably the most commonly used protein for wine fining, as it preferentially removes high molecular weight tannins [17,18,19]. Meanwhile, egg albumin (derived from egg whites) and casein (milk protein) [20] are the main animal proteins used for wine fining worldwide. However, egg and milk proteins are well-known food allergens, so their utilization poses a risk to allergic consumers if residues were to remain in the wine after the fining treatment [21,22,23,24]. To manage this risk, the EU introduced a regulation stating that egg and milk proteins have to be declared on wine labels if they are found in wine at a concentration greater than 0.25 mg/L [25]. These issues with animal proteins resulted in an increasing demand for wines elaborated without fining agents from animal sources, which is reinforced by the rising demand for vegetarian/vegan wines and for “natural” or “low intervention” wines. This has led to an increasing interest in exploring alternative wine fining agents (see Figure 1), especially in the past 20 years. One important research trend includes the assessment of the fining efficacy of proteins extracted from plant materials (e.g., from cereals, grape seeds, potatoes, legumes, etc.), as well as non-proteinaceous plant-based materials (e.g., cell wall polysaccharides and pomace materials). 

### 2.1. Wine Fining with Cereal-Based Proteins

#### 2.1.1. Gluten

Probably the first source of plant proteins that researchers have looked at to replace proteinaceous fining agents of animal origins is wheat gluten, an inexpensive, readily available and food grade material largely used as an ingredient in the food industry. Indeed, since the beginning of the 3rd millennia, several authors used wheat prolamins, commonly called gluten, as fining agents for both musts and wines. Gluten is a mixture of different water-insoluble proteins constituting the storage proteins of the wheat kernel, and comprises the monomeric alcohol-soluble gliadins and the insoluble polymeric glutenins. In these investigations, gluten has been modified in different ways (e.g., by hydrolysis and deamidation) in order to explore how its composition affected the ability to bind different wine components during fining trials, particularly when compared to other commercially available wine fining agents [5,6,13,26,27,28,29,30,31]. 

Studies on gluten focused mostly on red wines, but some were conducted on white and model wines. The first report on the use of gluten in fining was on a Burgundy red wine, and looked at the fining effectiveness of enzymatically hydrolyzed or deamidated glutens (five preparations in total) in comparison to egg proteins and gelatin [27]. The authors reported promising results, with large reductions in wine turbidity being achieved by the different gluten preparations (range 67%–86%), even if egg proteins performed in a better way for this aspect. Lees’ volume of gluten-fined wines was low, but was higher than gelatin, while no sensorial differences were detected among the wines treated with glutens and commercial fining agents. On the basis of these results, the authors concluded that wheat gluten protein preparations were effective fining agents for red wines. 

Another study compared glutens to gelatin for clarification of young red wines [5]. Gluten and gelatin were similar in reducing turbidity, but gluten had the advantage of producing less lees (−60%) and reduced the content of polyphenolic material less than gelatin did. 

A study conducted on Tempranillo red wines looked at the clarification ability of seven gluten preparations [13]. Some of the wines treated with glutens had residual wine post-filtration turbidity that was lower than that achieved using gelatin. The authors attributed this result to the higher isoelectric points and superficial charge densities of glutens in comparison with gelatin, in agreement with what already reported by others [32].

Other authors used four gluten preparations (two unmodified and two hydrolyzed wheat glutens) in fining both model and red wines. Results showed that, similarly to gelatins, gluten could selectively precipitate condensed tannins. Some of the gluten preparations were reported to remove highly galloylated tannins in similar quantities as gelatin, while gelatin was more effective in removing total tannins [28]. 

Another study suggested that gluten used as fining agent had less impact on the color and anthocyanin content of red wines, while the traditional animal based fining agents impacted more significantly the wine phenolic composition [33].

A paper by Granato et al. [10] looked at commercial wheat gluten proteins as fining agent in red wines at three ageing times (young, 12 and 24 months), and compared it with several other fining agents. The authors reported that gluten was very fast in lowering wine turbidity upon application, but also that, without racking the wine, the turbidity would increase again. Conversely to what the same authors observed in white wines, fining the red wines with gluten had a small impact on proanthocyanidins [30]. Gluten did not remove color and removed significantly less volatiles than gelatin from the wines.

On white wines the published research is less abundant than for reds, but is still sufficient to provide an insight on the mechanism of action of glutens. A study conducted on Chardonnay compared gluten to casein, gelatin-tannin association, fish glue, and bentonite [6]. Gluten could clarify the wine only at high addition rates (20 to 40 g/hL), while fish glue and casein were much more effective at low addition rates (1 and 5 g/hL, respectively). 

A study on Chardonnay and Gewürztraminer compared gluten fining with animal proteins [4]. Gluten was able to clarify only one of the two wines, confirming its low clarification ability for white wines [6]. In terms of volatiles, a trained panel found that gluten caused some significant modifications in the spicy and floral/honey flavor of the Chardonnay wine, even if the authors did not exclude a panelists effect and therefore suggested that more studies were needed to understand this occurrence. 

Finally, an article looked at the effect of hydrolyzed wheat gluten on three young white wines from Portugal [34]. Gluten showed a similar clarification ability to that of casein, PVPP and bentonite, while it produced a lower amount of lees (2.8%–3.7% depending on the addition rate).

It must be highlighted that gluten is a complex mixture made of tens of proteins and protein polymers [35], all of which are insoluble, and therefore the mixture is extremely difficult to prepare in a standardized manner. This is reflected in the results available in the literature, in which a full characterization of the used proteins is generally lacking. This means that gluten is generally processed before being used as fining agent, but also the extent of the modification due to the processing adopted affects its action on wine, as can be seen in the contrasting results reported above. 

Concerning the possible effects of the presence of gluten residues in fined wines, it needs to be considered that gluten proteins could cause different adverse reactions for people suffering from both IgE-mediated allergic reactions [36] and Celiac Disease (CD) [37]. In addition, recently a third type of reaction to wheat has been described named Non Celiac Gluten Sensitivity (NCGS), but the involvement of the gluten proteins for this disorder is still a matter of debate [38]. Therefore, wheat and related cereals (i.e., rye and barley) are included in the list of the allergens to be declared on food labels (Annex III) [39]. Nevertheless, the consumption of wines fined with gluten by CD patients is not likely to cause problems according to the opinion expressed by the European Food Safety Authority (EFSA) Panel [40].

However, also in this case the mode of preparation, and in particular the degree of hydrolysis could result in the production of soluble peptides remaining in the wine. While this is unlikely to be a problem for CD patients, who as stated by EFSA require a certain quantity of residue to trigger the adverse reaction, this is not the case for allergic people to whom the dose-eliciting reactions can be much lower. As a matter of fact, according to some studies [7,41], some residues can be present in gluten fined wines. Indeed, the EFSA Panel on Dietetic Products, Nutrition and Allergies (NDA) already stated in 2004 that “taking into account the level of wheat proteins reported to cause allergic reactions in severely allergic individuals, the panel considers that wines and musts treated with hydrolyzed wheat gluten could trigger an allergic reaction” [40].

Minimal information is available on the possible presence of gluten residues in wines, probably due to the limited diffusion of this practice in winemaking. Residues in gluten fined wines have been searched by an enzyme-linked immunosorbent assay (ELISA) and using a dot-blot method with chemoluminescence detection. The authors did not detect any residues in red wines treated with up to 20 g/hL of gluten and therefore concluded that, given that the limit of detection of the immunotechnique used was less than 1 mg/L, the gluten content of wines would have been lower than this, a level deemed sufficient by the EFSA that expressed a positive opinion on the use of gluten for winemaking [40]. Another study reported the presence of gluten residues in some white wines. It is noteworthy that, conversely to what was observed for other protein fining agents, in this case secondary treatments with bentonite, silica gel or tannin did not fully remove gluten residues [42]. Lately, residues of gluten in fined wines have been searched by immunoenzymatic analyses and liquid chromatography coupled to mass spectrometry [7]. The authors analyzed wines treated with different types of gluten fining added at different concentrations (1–300 g/hL), and found that immunological methods revealed gluten residues on wines treated with 50–300 g/hL, while by LC-MS residues were detected in wines fined with as low as 1 g/hL of gluten product. The authors suggested that adverse reactions against gluten treated wines cannot be excluded as some residual antigenicity (as detected by IgE binding with sera from wheat allergic patients) was detected in wines fined with partially hydrolyzed gluten, but not when the non-hydrolyzed product was used.

Despite these problems, and the fact that gluten was allowed for winemaking in 2004 [43], it is somewhat surprising that gluten did not receive the same attention by the OIV (International Organisation of Vine and Wine) of other allergenic proteins such as egg and milk in terms of labeling requirements. To the best of our knowledge, no gluten-based wine fining agents are currently present in the market.

#### 2.1.2. Maize Zeins and Rice Proteins

A plant protein investigated as an alternative fining agent is zein, a group of proteins constituting the prolamins of maize. Zeins have several attractive characteristics for their utilization in wine fining: they can easily be extracted from commercial corn gluten meal, the main by-product of the starch industry, and therefore can be purchased at a very low price. Moreover, maize is not among the cereals containing gluten and therefore does not need to be declared on the label [44,45], although some cases of allergy to corn have been reported [46].

Corn zeins were successfully extracted from commercial maize flour in both reducing (Na_2_SO_3_ as reducing agent) and non-reducing conditions. In a first experiment conducted on a red wine [8], the two zeins preparations were compared with two commercial gelatins (one non-hydrolyzed and one hydrolyzed). Only reduced zeins were able to decrease wine turbidity and remove phenolic compounds (e.g., anthocyanins and proanthocyanidins) in a dose-dependent way. The removal ranged between 6% for the unreduced zeins to 24% for the reduced one, indicating that protein reduction improves fining ability. In a follow up article, these results were confirmed in three Italian red wines. Additionally fining with zeins, although removing anthocyanins, did not significantly affect the wine color, nor did it negatively modify the sensorial properties when compared with the gelatin-fined wines [9].

Another plant source for wine fining could be rice, a recognized hypoallergenic material [47] which also is not required for allergenic labeling [45]. The rice grain is mostly made up of starch, and after its extraction a protein by-product is left, representing an interesting material that has been sporadically investigated as wine fining agent [48,49,50]. An early study looked at the chemical and sensory effects of six plant proteins including one from rice in both red and white wines [48]. Surprisingly, even possessing a very low proline content, which is generally recognized as a factor affecting the protein-tannin interaction, rice proteins showed better clarification ability than gelatin, without inducing modification in both the composition and sensory attributes of the wines. A second investigation showed that rice protein extracts had the ability to clarify wines similarly to caseinate in white and rosé wines and to gelatin in red wine, while reducing white wine oxidability, without inducing detectable sensory modifications. Additionally, rice proteins showed a high ability to remove tannins and to decrease astringency in red wines [49]. In contrast, a low impact on wine chemical composition by rice fining was shown in a recent study [50], in which the fining ability of rice proteins in red wine was compared to that of gelatin, PVPP and some other plant-based proteins. Rice proteins only marginally reduced wine astringency, but had even lower impacts on the wine color. All this variability can be attributed to the different modes of preparation of the protein extracts, which can affect the proportions among the different components of the rice proteins.

Despite being promising, maize and rice proteins are still not commercially available and therefore more research and development is required.

### 2.2. Wine Fining with Legume-Based Proteins

Proteins from pea, soybean, and lentil have also been tested for their fining efficiency against commercial standards as K-caseinate and gelatins. In a recent study the fining ability of several legume-based proteins (soy protein, lentil flour, wheat gluten, and 3 pea protein preparations) was compared to that of a gelatin on a red wine at 3 levels of ageing (young, 12 months old and 24 months old) [10]. The authors showed that plant proteins were as effective as gelatin in fining, with each plant proteins having different impact on some specific wine traits. Interestingly, the authors reported that in comparison with gelatin, fining young wines with plant proteins had a lower impact on fermentation aroma compounds. All of the fining treatments did not alter the color tonality of the wines, despite the ability of some legume proteins as soybean, lentil and pea hydrolysates to remove some anthocyanins. A key finding of the study was that a limited protein hydrolysis greatly modified the fining ability of pea proteins, a fact that could be used to tailor certain protein preparations to the needs of different wine styles.

Another study assessed the fining ability of soybean and pea proteins to reduce astringency in red wine compared to commercial gelatin and PVPP. Results showed that pea and soy proteins had similar ability in removing tannins and consequently astringency than PVPP, but quite less than gelatin [50]. 

Other authors studied the potential of insoluble protein isolates from pea, lentil, and soybean as fining agents in model and white wine [30]. All legume proteins were effective in speeding up clarification. Lentil proteins were the best in removing monomeric and dimeric flavonols, even though they had the highest impact in removing fermentation aroma compounds, while for pea and soy proteins the aroma reduction effect was similar to that of commercial fining agents.

Pea proteins were compared to PVPP and K-caseinate for white wine fining [51]. Results showed that flavonoid and non-flavonoid phenols significantly decreased in all wines treated with the three fining agents, as well as wine color, while only K-caseinate reduced the wine browning potential. Pea protein and K-caseinate were considered the best for wine clarification, while from a sensory point of view all fining agents had similar effects on wine.

All of these studies agree in indicating legume proteins as a viable source of fining agents for winemaking, as confirmed by the large number of pea-based commercial fining agents available in the market. Generally the recommended doses for legume-based commercial fining agents range between 5 to 30 g/hL, and this depends on the vinification stage and on the type of wine to be treated.

Concerning the allergenic aspects of fining wines with legume proteins, it must be noted that soybean, lupine and peanuts but not pea proteins are present in the list of allergenic substances of the Annex III of the EU Directive [45]. In addition, pea proteins are approved by the OIV as wine fining agents [43], and several commercial products are available. Pea allergy is infrequent, but the immunological cross-reactivity among legumes is well documented [52,53], indicating that pea proteins can be allergenic for some people sensitized to peanut, soy or lupine (all included in the list), because proteins of these legumes share IgE epitopes [54]. Given that cross-reactivity among legumes can be a source of risks for allergic consumers, it is somewhat unexpected that the regulatory bodies allowed these proteins as fining agents in wine without restrictions (e.g., labeling requirements, mandatory detection of residues). 

To the best of our knowledge the only legume studied for the presence of residues in wine is lupine. However, in this case no detectable residues were found in red wines and those possibly remaining in white wines were eliminated by bentonite treatment [41]. Even if these findings indicate that lupine is not likely to represent a risk, it would also be worth deepening this aspect for the other legumes. 

### 2.3. Wine Fining with Potato Proteins

Proteins from potatoes can also be used as fining agents in wine. In particular, patatin P, a major 39–45 kDa glycoprotein from potatoes, represents over 40% of the soluble proteins of this tuber [55]. Several processing procedures for potatoes produce aqueous by-products rich in this soluble protein that can be recovered (e.g., by ethanol precipitation [56]) and used for different applications. Recently, patatin has been studied for its potential use as wine fining agent, and in particular for its ability to bind wine phenolic compounds and thus reduce astringency [11]. This study, conducted on Aglianico red wine, compared patatin with other commercial fining agents (K-caseinate, gelatin, egg albumin). Despite removing some anthocyanins, patatin did not deplete the color of the wine, while it significantly reduced the content in total phenolics and tannins, with a reduction in astringency similar to that achievable with gelatin, and better than those obtained with the other commercial fining agents [11].

Patatin was also tested as a must fining agent in two white varieties, and its efficacy compared to that of bentonite and K-caseinate [12]. Patatin reduced the browning potential of the musts more than K-caseinate and lowered the turbidity of the must during settling thanks to its good flocculating activity. 

The fining ability of several plant proteins including a commercial patatin was studied for astringency reduction in red wine in comparison with gelatin and PVPP [50]. Results showed that, at the same dosage, patatin was the second most effective fining agent after gelatin in removing tannins and total phenolics. In contrast with the results of Gambuti and colleagues [11], a commercial patatin-based fining agent was found to significantly modify wine color as measured by CIELab, suggesting the need to be cautious when using this product for fining wine. From the sensory point of view both patatin and gelatin significantly reduced wine astringency [50], in agreement with previous studies [11], confirming the ability of patatin to replace gelatin as fining agent for astringency reduction in wines. 

Potatoes rarely induce allergies, in spite of the high levels of consumption [39,57], although it has been identified as a major cross-reactive protein in the latex-associated potato allergy, and appears to be involved in atopic dermatitis [58]. To the best of our knowledge, no published data are available on the presence of patatin residues in wines. Given the low allergenicity of potatoes, the risk of having an adverse reaction after consuming wine fined with patatin is very limited, and patatin has already been authorized by the OIV with the resolution OIV-OENO 495-2013 [59]. 

Since patatin shows excellent fining properties, can be easily recovered from by-products of the potato’s processing, and is approved by the OIV [59], commercial products based on potato proteins have been developed and are now available for winemakers. Generally the recommended doses for these commercial fining agents range between 1 to 20 g/hL depending on the vinification stage and on the type of wine to be treated.

### 2.4. Wine Fining with Grape Seed Extract (GSE)

One of the main avenue of investigation concerns the preparation of fining agents sourced from materials that are naturally present in grapes [60,61], wine or yeasts [34,62,63]. Obviously, as these compounds are already present in every wine, they do not need to be declared. One of the most promising products in this category can be obtained from grape seeds.

Grape seeds recovered from the vinification process constitute a by-product of the winemaking process that is often used by the food industry to extract edible oil. As a result of this industrial process a dry residue (grape seed flour) mostly made up of fiber, phenolic compounds, polysaccharides, and proteins is obtained. The notion that this residue contains a good amount of proteins has been exploited to develop a new protein-based fining agent made solely of materials endogenous to grape, which was named Grape Seed Extract (GSE) [14,15]. A quick and inexpensive extraction and clean up procedure allows to obtain an extract rich in proteins and suitable as fining agent for wine. After testing several extraction conditions, Vincenzi et al. [15] developed a method that consisted of dissolving the grape seed flour in an alkaline solution (pH 10.5) and, after 3 h of extraction, separating the liquid phase by settling. Subsequently, the proteins of the liquid phase were made insoluble by acidification to pH 3.0, and the resulting acid-precipitated material was recovered by centrifugation, dissolved in 0.01 N NaOH, and stored at 20 °C. This procedure yielded a GSE containing over 40% of protein. It is worth noting that the same procedure can also be applied starting from whole grape seeds after milling and defatting with hexane (unpublished). The GSE extraction procedure and utilization has been patented [64].

Initial fining trials made on model solutions spiked with commercial grape seed tannins and on a Cabernet Sauvignon wine indicated that GSE was able to significantly lower astringency [15] as measured in vitro by the Astringency Mucin Index [65] and, in vivo, by sensory analysis.

Further experiments were made to compare the wine fining efficacy of GSE with that of commercial fining agents, including patatin, pea proteins, polyvinylpolypyrrolidone (PVPP) and K-caseinate in a white and a rosé wine, and ovalbumin and gelatin in a red wine [14]. Fining trials were made on wines with GSE addition rates within the range of those suggested for the other commercial fining agents used (5–20 g/hL). Data showed that GSE performed well for different wine styles. In particular, it significantly decreased wine turbidity, with dose-dependent reductions ranging from −10% (for red wine at 5 g/hL) to −67% (for white wine at 20 g/hL). GSE showed little effect on wine chromatic characteristics and oxidative stability, independently of the rate used, while it significantly reduced the proanthocyanidin content in the red wine. Compared to ovalbumin, the sensory effect on astringency was significant, and was obtained using a dosage of GSE that was three times lower. In general, GSE was shown to have positive effects on wine turbidity, color, oxidative stability and improvement of sensorial properties, confirming its efficacy as fining agent [14,15].

Within this scenario, GSE has been proposed as the first effective fining agent endogenous to grapes that should not be subjected to the legal restrictions for the presence of allergenic substances in wine, nor investigated for the presence of residues in wine. From this point of view, using this fining agent will go towards the well-established trend of consumers seeking more natural wines that are produced without the addition of foreign substances. However, despite its demonstrated fining effectiveness, GSE is not yet commercially available as it needs to be approved by the OIV.

## 3. How to Avoid the Allergenic Risk Deriving from Exogenous Protein Residues in Wines?

In the case of wine, the presence of hidden allergens or residues of allergenic materials used for fining has to be considered as a possibility, and this has to be taken into account by wine producers starting from 2012, when they were obliged to indicate on the label the presence of milk and milk-based products and eggs and egg-based products when these materials are present (i.e., can be detected) in quantities higher than 0.25 mg/L in the final product [25], as measured by the OIV recommended ELISA methods [66]. Therefore, in order to fulfill the labeling requirements, any wine treated with the above-mentioned fining agents must be analyzed for their presence before bottling and, in the case of positive results, an indication of the presence of egg or milk proteins in the wine must be reported when the content of milk or egg proteins is above 0.25 mg/L. However, at the moment this restriction only applies to egg and milk proteins, whereas no regulation exists for other types of foreign substances that can be found in wine. Taking into account that the list of allergenic materials is continuously revised, it is possible that other allergenic proteins, including those derived from plants, will be considered by the regulatory bodies.

The OIV published a resolution [67] containing clear guidelines for good fining practices that, when adopted, will reduce the risk of having protein residues in wines, independently of the origin of the protein material used as fining agent. By applying these guidelines, winemakers can limit the presence of protein residues in wines, thus fulfilling the duties of the current regulation.

Even if these guidelines are developed with reference to casein and egg white, they are also likely to be effective for other proteins including those of plant origin in case their potential allergenicity will have to be taken into consideration.

The full guidelines can be found in the OIV resolution [67], but generally speaking the suggestions included: (i) ensuring the purity and integrity of the fining agents used, (ii) executing small-scale laboratory fining trials representatives of the treatment conditions to be used in the winery, (iii) using as little water as possible during the preparation of the fining agents as well as, (iv) using the smallest amount of fining agent required to attain the desired result, (v) mixing adequately the fining agent with the juice/wine to be treated and allow enough contact time before removing the insoluble material formed, (vi) using best practice filtration methods to remove insoluble protein or fining agents and analyze wines to confirm that no residues are detected, (vii) checking the presence of residues at every winemaking step in representative samples in order to, on one hand be able to take appropriate corrective actions where the analysis of such wines indicates the presence of residual fining agents, and on the other hand to include in wine labels the recommended wording or pictograms indicating the potential presence of allergens [25].

These steps outlined in the OIV resolution were tailored to limit the content of fining agents with allergenic potential such as casein and egg white that are required to be listed on wine labels. Alongside these guidelines, several authors have demonstrated the efficacy of secondary fining with bentonite for the removal of residual proteinaceous processing aids, including those originating from egg [21], milk [68,69], fish [70], but also from plant sources as lupine and wheat gluten [41,42]. This suggests that coupling the two treatments (filtration and bentonite addition) is likely to be the best practice to adopt when proteinaceous fining agents are used.

However, following these guidelines can certainly represent an extra cost in terms of number of analysis needed, labor, time and paperwork, so that winemakers would welcome alternative methods to solve the problem of the presence of exogenous protein material in wines. The most logical alternative would be to use proteinaceous material already present in grapes and wines as fining agents, which obviously cannot be considered as a foreign substance.

Indeed, these types of products, including proteins extracted from both grape seeds (GSE, see above) and yeasts (yeast protein extract, YPE, commercially available) have been recently developed to achieve effective fining while not introducing any risk due to hidden allergens of foreign origin [5,13,14,15].

Since the first report showing that apple cell wall material had high affinity for tannins [71], several investigations have looked at cell wall material (polysaccharides, pomace and fibers) from different plant sources as a novel wine fining agent [60,72,73,74,75], while encouraging tannin reduction to be reported. One of these materials comes from the grape skins, and is mainly constituted of insoluble cell wall polysaccharides which have been confirmed to have an affinity for tannins and then to be suitable for their removal from the wine [61,75,76]. Similarly to what was stated for GSE and YPE, if proven effective, the use of grape skin extracts for wine fining would also avoid all of the issues linked to the potential allergenicity of proteins (both animal and plant-based).

## 4. Development of Novel Plant Protein Fining Agents

**Figure 2:**
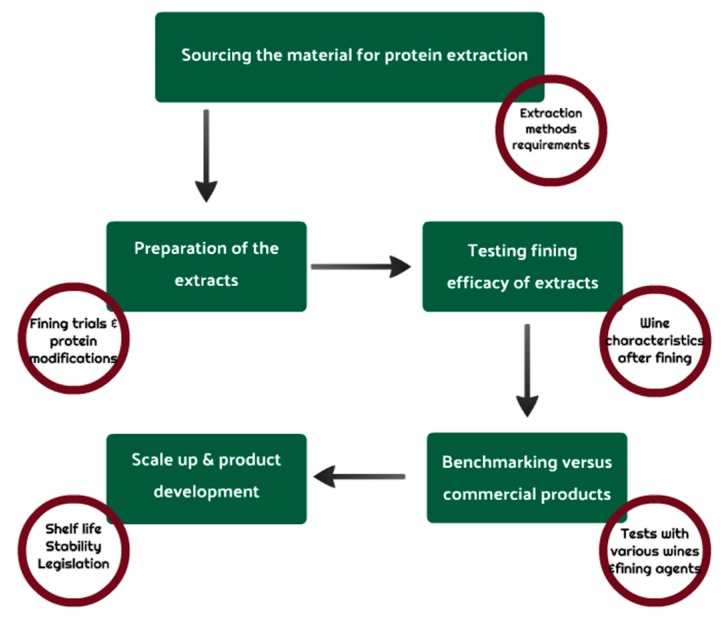
Schematic representation of the key steps required for the development of novel plant protein fining agents.

By looking at the articles proposing proteinaceous fining agents or testing some that have already been developed, it is possible to find a common approach for the development and evaluation of the fining performances of novel fining agents. This is summarized below and reported schematically in Figure 2.

- Sourcing the material for protein extraction. The development of a new fining agent needs to begin with the selection of the starting material (protein source) from which extracting the proteins. Several considerations can be made to inform this decision. Ideally, the source should be rich in proteins, and could be both an intact plant organ (e.g., seeds, fruits, roots etc.), or a by-product resulting from an industrial plant processing. Several examples have been previously discussed, including by-products of the oil (e.g., the grape seed flour [15]) and of the starch (e.g., maize zeins [8], rice proteins [49]) industries. Ideally, the selected material should be inexpensive and readily available, because otherwise the products, even if shown to be effective, would be inappropriate for large scale distribution. Additionally, the starting material should be allergen free, or at least not subjected to allergenic labeling [45], safe for consumption, and containing no or very low quantities of compounds that would potentially impact the wine sensory attributes (e.g., off flavors, colored material, phenolics, etc.).
- Extraction methods requirements. Proteins can be extracted from plant materials with a variety of methods, but in this case protein extraction needs to be performed only with the use of food grade and inexpensive reagents, and should include only a limited number of steps in order to be as simple as possible and consequently economically viable.
- Preparation of the extracts. The different extraction methods theoretically designed, also considering the solubility properties of the protein fractions of interest, will need to be tested for protein content (e.g., by nitrogen quantification or colorimetric methods) in order to obtain the highest yield. Based on the extraction yield, the best performing method is then selected. The liquid extracts obtained can be kept in a liquid form when the extracted proteins are soluble, or the proteins can be partially purified (e.g., by precipitation via ethanol addition or pH changes), but the purification steps should be reduced at the minimum due to their additional direct (e.g., reagents, equipment, time, labor), and indirect (e.g., loss in protein yield) costs. A preliminary fining test (see next point) at this stage can be done to inform about the potential efficacy of the extract. Proteins can also be modified to improve their binding ability. These modifications generally include protein thermal denaturation [8], partial hydrolysis, and use of food grade reducing agents (e.g., sulfites). All these treatments are performed to modify the size and number/accessibility of the tannin binding sites, so to favor the interactions with wine phenolics compounds [6,77].
- Testing the fining efficacy of the selected extract. The fining ability of the obtained proteinaceous extract(s) will need to be assessed on un-fined musts and/or wines. Preliminary fining trials should be done using different dosages of the fining agent at laboratory scale (e.g., 100–200 mL of must or wine). To this aim, the chosen dosages will depend on the protein content of the extract, but generally a preliminary trial could look at addition rates of 0, 1, 5, 10, 20 and 40 g/hL. If the extract is in a solid form, it is recommended to prepare a concentrated stock solution by dissolving/dispersing it in a small quantity of liquid (e.g., water, must or wine). The fining agent will then be added to the must or wine in a graduated cylinder and mixed well. For the assessment of chemical and sensory impact of the treatment, the wine can be let in contact with the fining agent for at least 24 h, while longer periods (e.g., 5 days) are needed to evaluate its clarification ability. During the contact period the volume of the precipitated material will be recorded to have information on how much wine is trapped on the lees [78] and on the compactness of the deposit, as this can affect the wine racking and/or filtration efficiencies. Variations in turbidity of the wine as a function of the different dosages used will also be recorded by nephelometry. This allows to have an idea of the time required to reach satisfactory wine clarification for the different doses. The wine will then be separated from the insoluble material by centrifugation (e.g., >3000 g) and/or filtration (e.g., glass fiber filter followed by 0.45 µm), and analyzed in comparison with the un-fined control sample (0 g/L fining agent). Wine chemical characterization should be performed according to the OIV official methods of analysis of reference [79], and should include basic wine parameters (e.g., pH, ethanol content, titratable acidity), chromatic characteristics, oxidative stability, phenolic content (e.g., by Folin-Ciocalteau method), and if more information is needed additional analysis as phenolic profile including anthocyanins for reds (by HPLC) [50,73], astringency assessments (e.g., mucin index [65]), variation in sensory attributes (e.g., astringency, bitterness, aroma [4,9]), and volatile composition by GC [10] can be done. Additionally, in case the starting plant material is already included in the list of the allergens, tests for checking the presence of protein residues (e.g., by immunological or MS-based methods) in the filtered wines treated with different doses are also necessary [80].
- Benchmarking versus commercial products. When a full analytical picture is obtained, the best fining addition rate(s) should be selected, and the experiment should be replicated benchmarking the new product with other commercially available fining agents. At times the benchmarking can be done simultaneously with the previous step, although this approach could be more time consuming. To gain information on the general effects of the new preparation, different wine styles should be tested, including at least one white and one red wine. For each wine, the effects must be considered in relation to the desired aims (e.g., clarification, stabilization, modification of the sensory properties) as they can be different depending on the wine characteristics. In this context, it could advisable to use the fining agents that are commonly employed for a specific type of wine as a benchmark.
- Scale up. Once the benchmarking has given satisfactory results, the experiment should be scaled up in terms of volume of wine treated (for example 100 L or more) to allow for more complex sensory tests requiring an amount of wine higher than that of the laboratory scale trials (e.g., descriptive sensory analysis and preference tests) and also to confirm the results obtained by these trials.
- Development of a commercial product. The product will need to be checked for stability, and its shelf life and appropriate storage conditions determined. Finally, authorization for use in winemaking will need to be requested to the appropriate governing bodies (e.g., the European Union, OIV).

Clearly the above listed guidelines are very general and will need to be adapted according to the specific characteristics of the material used, the aims of the fining trial and the type of must/wine to be treated. Nevertheless, these guidelines can be considered as a good starting point for those approaching the development of a new plant-based fining agent.

## 5. Conclusions

The novel solutions proposed and currently investigated for fining wines with plant materials are drawing attention from suppliers of oenological products, wine producers and consumers for different reasons, including safety, personal preferences and economic convenience. Fining agents based on plant proteins continue to be studied and benchmarked versus animal counterparts, and this has led to the development of several products (e.g., from pea and potato) that have already been approved, commercialized and successfully used by winemakers worldwide. This has certainly solved some of the issues that led to the proposal of plant fining agents in the first place, such as the lack of the labeling requirements necessary for allergenic materials, and provides a response to the growing consumer requests for more “natural wines” and wines that are vegetarian/vegan friendly. However, as discussed above, plant proteins can also be allergenic, and given that the list of allergens is going through periodic reviews, it is possible that some of these proteins will end up being included in this list in the future, so the same problem faced with animal-based proteins could arise. Research in wine fining with plant proteins should continue to develop and test the efficacy of different preparations, but also to more systematically study the potential presence of residues in wines due to their potential allergenicity. Therefore, it seems advisable that future research efforts should be more directed towards the development of solutions based on materials endogenous to wine or grapes as grape seed and yeast protein extracts, pomace, insoluble cell wall material and fibers, all products that are naturally present in wines and therefore should not raise the concerns deriving from the presence in wine of substances of foreign origin.
